# Supplementary material for: Inhibition of Na+/K+ ATPase blocks Zika virus infection in mice
Source: Commun Biol. 2020 Jul 15;3:380. doi: 10.1038/s42003-020-1109-8 (PMC7363852; doi:10.1038/s42003-020-1109-8)
Supplement: Supplementary file 1 — Description of Additional Supplementary Files [file 42003_2020_1109_MOESM1_ESM.pdf]

Description of Additional Supplementary Files

The source data in the Supplementary Data 1 are described below.

Figure 1:

The source data of antiviral effects of ouabain and digoxin (The main graphs, Figure 1a-h and k), we displayed the viral titer and amount of RNA, statistical analysis was made between control and drug group.

Figure 2:

The source data of ouabain and digoxin block viral RNA synthesis (Figure 2a and b), we displayed the viral titer and RNA synthesis, statistical analysis was made between control and drug group.

Figure 3:

The source data of ZIKV inhibition with ouabain and digoxin via Na^+/K^+-ATPase (Figure 3a-d), we displayed the antiviral effect of ouabain and digoxin (the percent of viral titer) in different concentration of Na^+/K^+ , statistical analysis was made in the indicated groups.

Figure 4:

The source data of ouabain decreases ZIKV viral load in adult mouse brain (The main graphs, Figure 4a), we displayed the viral titer and significance between vehicle and ouabain group.

Figure 5:

The source data of effect of ouabain treatment in maternal and fetal tissues (The main graphs, Figure 5d-g), we displayed the source data of fetus survival, fetus size, the viral RNA of fetal head and placenta, statistical analysis was made between vehicle

and ouabain group.
