# Supplementary material for: Inhibition of Na+/K+ ATPase blocks Zika virus infection in mice
Source: Commun Biol. 2020 Jul 15;3:380. doi: 10.1038/s42003-020-1109-8 (PMC7363852; doi:10.1038/s42003-020-1109-8)
Supplement: Supplementary file 3 — Reporting Summary [file 42003_2020_1109_MOESM3_ESM.pdf]

nature research

Corresponding author(s): Wei Wang

Last updated by author(s): Jun 18, 2020

Reporting Summary

Nature Research wishes to improve the reproducibility of the work that we publish. This form provides structure for consistency and transparency in reporting. For further information on Nature Research policies, see our Editorial Policies and the Editorial Policy Checklist.

Statistics

For all statistical analyses, confirm that the following items are present in the figure legend, table legend, main text, or Methods section.

n/a	Confirmed	
☐	☒	The exact sample size (*n*) for each experimental group/condition, given as a discrete number and unit of measurement
☐	☒	A statement on whether measurements were taken from distinct samples or whether the same sample was measured repeatedly
☐	☒	The statistical test(s) used AND whether they are one- or two-sided<br>*Only common tests should be described solely by name; describe more complex techniques in the Methods section.*
☒	☐	A description of all covariates tested
☒	☐	A description of any assumptions or corrections, such as tests of normality and adjustment for multiple comparisons
☐	☒	A full description of the statistical parameters including central tendency (e.g. means) or other basic estimates (e.g. regression coefficient) AND variation (e.g. standard deviation) or associated estimates of uncertainty (e.g. confidence intervals)
☐	☒	For null hypothesis testing, the test statistic (e.g. *F*, *t*, *r*) with confidence intervals, effect sizes, degrees of freedom and *P* value noted<br>*Give P values as exact values whenever suitable.*
☒	☐	For Bayesian analysis, information on the choice of priors and Markov chain Monte Carlo settings
☒	☐	For hierarchical and complex designs, identification of the appropriate level for tests and full reporting of outcomes
☒	☐	Estimates of effect sizes (e.g. Cohen's *d*, Pearson's *r*), indicating how they were calculated

Our web collection on statistics for biologists contains articles on many of the points above.

Software and code

Policy information about availability of computer code

Data collection	Vectra ss, Perkin Elmer
Data analysis	GraphPad Prism 7 , Origin software.

For manuscripts utilizing custom algorithms or software that are central to the research but not yet described in published literature, software must be made available to editors and reviewers. We strongly encourage code deposition in a community repository (e.g. GitHub). See the Nature Research guidelines for submitting code & software for further information.

Data

Policy information about availability of data

All manuscripts must include a data availability statement. This statement should provide the following information, where applicable:

- Accession codes, unique identifiers, or web links for publicly available datasets
- A list of figures that have associated raw data
- A description of any restrictions on data availability

All data supporting the findings of this study are available within the paper and its supplementary files or available from the corresponding author upon reasonable request. Source data are available in Supplementary Data 1.

Field-specific reporting

Please select the one below that is the best fit for your research. If you are not sure, read the appropriate sections before making your selection.

☒ Life sciences ☐ Behavioural & social sciences ☐ Ecological, evolutionary & environmental sciences

For a reference copy of the document with all sections, see nature.com/documents/nr-reporting-summary-flat.pdf

Life sciences study design

All studies must disclose on these points even when the disclosure is negative.

Sample size

As shown in the results of all mice experiment, we use Student's t test for each analysis.

Ouabain administration to ZIKV-infected adult mice:
The infected mice were i.p. administered with 2 mg/kg ouabain (n = 13) and a vehicle control (n = 12).

Ouabain administration to ZIKV-infected pregnant mice.
For mating, 6- to 7-week-old male and nulliparous female mice were co-housed and pregnant mice were randomly divided into two groups. 14 pregnant dams number of animals in each experimental group, 2 to 4 pregnant dams for each group. placentas and fetuses were harvested from the infected mice and ZIKV RNA levels were determined by qRT-PCR, 2 to 4 individuals were randomly selected from each pregnant dam.

Data exclusions

No data were excluded from the analyses.

Replication

Our animal experimental procedures were carried out according to ethical guidelines and were approved by the Animal Care Committee of Wuhan Institute of Virology (Permit Number: WIVA25201801) to verify the reproducibility of the experimental findings.

Ouabain administration to ZIKV-infected adult mice:
Six- to seven-week-old Ifnar1-/- mice were assigned randomly to two groups and infected by the i.p. route with 1 × 10*4PFU ZIKV (H/PF/2013). Three hours later, the infected mice were i.p. administered with 2 mg/kg ouabain (n = 13) and a vehicle control (n = 12), respectively. Treatments were consecutively administered once a day for up to 5 days. The viral burden of brain, serum was measured by plaque assay. Brain sections were further stained with anti-ZIKV NS3 rabbit serum, glial fibrillary acidic protein (GFAP; Dako), and DAPI.

Ouabain administration to ZIKV-infected pregnant mice:
Pregnant mice were randomly divided into two groups ,and were intragastrically administered ouabain at 3 mg/kg of body weight, or vehicle control every day. Our experiment performed as shown in the flow chart of the manuscript.

Randomization

All mice were assigned randomly to experimental groups. The mice in the different experimental groups were treated with ouabain depending on the regular time of day.

Blinding

Ouabain administration to ZIKV-infected adult mice:
The infected mice were i.p. administered with 2 mg/kg ouabain (n = 13) and a vehicle control (n = 12).

Ouabain administration to ZIKV-infected pregnant mice:
For mating, 6- to 7-week-old male and nulliparous female mice were co-housed and pregnant mice were randomly divided into two groups. 14 pregnant dams number of animals in each experimental group, 2 to 4 pregnant dams for each group. placentas and fetuses were harvested from the infected mice and ZIKV RNA levels were determined by qRT-PCR, 2 to 4 individuals were randomly selected from each pregnant dam.

Reporting for specific materials, systems and methods

We require information from authors about some types of materials, experimental systems and methods used in many studies. Here, indicate whether each material, system or method listed is relevant to your study. If you are not sure if a list item applies to your research, read the appropriate section before selecting a response.

Materials & experimental systems

n/a	Involved in the study
	☒ Antibodies
	☒ Eukaryotic cell lines
☒	Palaeontology and archaeology
	☒ Animals and other organisms
☒	Human research participants
☒	Clinical data
☒	Dual use research of concern

Methods

n/a	Involved in the study
☒	ChIP-seq
☒	Flow cytometry
☒	MRI-based neuroimaging

Antibodies

Antibodies used

ZIKV NS3 ; DyLightTM 488 labeled goat anti-rabbit IgG; anti-Ifnar antibody;primary antibodies cytokeratin ;Sox2; Secondary antibody

Validation

anti-ZIKV NS3 (gifted from Dr. Andres Merits, University of Tartu, Estonia);
DyLightTM 488 labeled goat anti-rabbit IgG (KPL, Gaithersburg, MD, USA);
anti-Ifnar antibody (MAR1-5A3, purchased from Leinco Technologies, Inc.) ;
primary antibodies cytokeratin (1:100, Abcam ab52625);
Sox2 (1:100, Mouse, Abcam ab92494);
Secondary antibody (1:400,Affinity Biosciences)

Eukaryotic cell lines

Policy information about cell lines

Cell line source(s)

ATCC

Authentication

The cell lines were authenticated.

Mycoplasma contamination

All cell lines tested negative for mycoplasma contaminatiom.

Commonly misidentified lines
(See ICLAC register)

No misidentified cell lines used in the study.

Animals and other organisms

Policy information about studies involving animals; ARRIVE guidelines recommended for reporting animal research

Laboratory animals

Ouabain administration to ZIKV-infected adult mice:
Mice with knockouts in IFNα and β receptors are highly susceptible to ZIKV infection and display relevant signs of disease. To evaluate whether ouabain protects against ZIKV infection in vivo, Ifnar1-/- (type I interferon (IFN) signaling knockout line) mice were treated with 10*4 plaque-forming units of ZIKV stain H/PF/2013 with 2 mg/kg ouabain.

Ouabain administration to ZIKV-infected pregnant mice:
IFN-α/β signaling pathway inhibits ZIKV infection in mice, and pre-treatment of anti-Ifnar antibody (MAR1-5A3) restated ZIKV sensitivity in mice. Therefore, C57BL/6 background pregnant mice treated with anti-ifnar antibody could be used to simulate pregnant women infected with ZIKV.

Ouabain administration to ZIKV-infected adult mice:
Details of the animals used:
Species: mice
Strain: Ifnar1-/- mice
Sex: male
Developmental stage:
mean age: Six- to seven-week-old
mean weight: 18 g
The source of animals: Adult male Ifnar1-/- mice (Six- to seven-week-old) were kept in the Laboratory.
Genetic modification status: Mice with knockouts in IFNα and β receptors.
Health/immune status: Healthy
Test: Specific-pathogen-free.

Ouabain administration to ZIKV-infected pregnant mice:
Details of the animals used:
Species: mice
Strain: C57BL/6 mice
Sex: female

Developmental stage:
mean age: 6- to 7-week-old pregnant female mice
mean weight: 25 g
The source of animals: 6- to 7-week-old pregnant female mice were kept in the Laboratory.
Genetic modification status: Mice were treated with anti-Ifnar antibody (MAR1-5A3).
Health/immune status: Healthy
Test: Specific-pathogen-free.

Wild animals

The study did not involve wild animals.

Field-collected samples

Housing:
type of facility: specific pathogen free
type of cage: IVC
bedding material: Sterilization shavings
number of cage companions: Fewer than five mice.
Husbandry conditions:
Light/dark cycle: 12 h light/12 h dark cycle
temperature: 25℃
Welfare-related assessments: We obeyed the laws and regulations for animal welfare protection.

Ouabain administration to ZIKV-infected adult mice:
How:
drug formulation and dose: Drug dissolved in normal saline and 2mg/kg/day.
site and route of administration: Mice were infected by the i.p. route with 1 × 10*4 PFU ZIKV (H/PF/2013). Three hours later, the infected mice were i.p. administered with 2 mg/kg ouabain and a vehicle control, respectively. Treatments were consecutively administered once a day for up to 5 days.
anaesthesia and analgesia: isoflurane
equipment and supplier: RWD lifescience
When: At 9 am every morning
Where: In the Laboratory Animal Center of Wuhan Institute of Virology, CAS (Wuhan, China)
Why: After repeated trials, 2 mg/kg was found to be non-toxic and harmless for Ifnar1-/- mice.

Ouabain administration to ZIKV-infected pregnant mice:
How:
drug formulation and dose: Drug dissolved in normal saline and 3mg/kg/day.
site and route of administration: The infected mice were intragastrically administered ouabain at 3 mg/kg of body weight, and vehicle control every day for 5 days post-infection.
anaesthesia and analgesia: isoflurane
equipment and supplier: RWD lifescience
When: At 9 am every morning
Where: In the Laboratory Animal Center of Wuhan Institute of Virology, CAS (Wuhan, China)
Why: After repeated trials, 3 mg/kg was found to be non-toxic and harmless for pregnant dams.

Ethics oversight

All animal experimental procedures were carried out according to ethical guidelines and were approved by the Animal Care Committee of Wuhan Institute of Virology (Permit Number: WIVA25201801).

Note that full information on the approval of the study protocol must also be provided in the manuscript.
